# A new species of the genus *Cenocorixa* (Hemiptera, Heteroptera, Corixidae) from China

**DOI:** 10.3897/zookeys.1055.63567

**Published:** 2021-08-06

**Authors:** Tong-Yin Xie, Guo-Qing Liu

**Affiliations:** 1 College of Agriculture, Northeast Agricultural University, Harbin 150030, China Northeast Agricultural University Harbin China; 2 Institute of Entomology, Nankai University, Tianjin, 300071, China Nankai University Tianjin China

**Keywords:** *
Cenocorixa
*, China, Corixidae, Heteroptera, Nepomorpha, new species

## Abstract

A new species of the genus *Cenocorixa* Hungerford (Hemiptera, Heteroptera, Corixidae) is described from China. *Cenocorixayuanjiangensis***sp. nov.** is reported from Yunnan Province. An updated key to the Chinese species of *Cenocorixa* is presented.

## Introduction

The genus *Cenocorixa* was erected by Hungerford in 1948 and currently contains 11 species. Usinger (1956) provided a key to the Californian (USA) species based on male characters. Jansson (1972) revised the genus, provided a revised key to males, and suggested that stridulation is important for the reproductive isolation of species. Stonedahl and Lattin (1986) reported six species of this genus from Oregon and Washington states (USA). The genus *Cenocorixa* was widely distributed in the western United States and Canada (Hungerford 1948; Brooks and Kelton 1967; Jansson 1972). Ren and Zhu (2010) subsequently reported three species of this genus from Yunnan Province, China.

In the present paper, we add one new species, *Cenocorixayuanjiangensis* sp. nov. With the addition of this new species, the genus *Cenocorixa* now contains four species in China and eight species in the United States and Canada (GBIF 2021).

## Materials and methods

The specimens were obtained from the Institute of Entomology, Nankai University (**NKU**), Tianjin, China. All specimens were cleaned in an ultrasound cleaner. The antennae were dissected, dried in ethanol, mounted, and sputtered with gold or chromium. The whole specimens and prepared antennae were observed with the use of a Hitachi S-3400N scanning electron microscope in the microscopy laboratory of the large scale instrument and equipment sharing service platform of Northeast Agricultural University, Harbin. Photographs were taken with a camera mounted on a Zeiss Discovery V20 microscope. All specimens studied are deposited in the Institute of Entomology, Nankai University (**NKU**), Tianjin, China.

## Taxonomy

### Key to the species of *Cenocorixa* from China

**Table:** 

1	Pala with distinct ridge across face, bearing a curving peg row consisting of 2–35 pegs (Fig. 3A–C)	**2**
–	Pala with inconspicuous ridge across face, bearing a curving peg row consisting of 28 pegs (Fig. 2A)	***Cenocorixayuanjiangensis* sp. nov.**
2	Pala with peg row continuous at transversal oblique ridge	**3**
–	Pala with peg row separated (interrupted) at transversal oblique ridge, containing13 anterior and 16 posterior pegs (Fig. 3C, F)	*** Cenocorixa montana ***
3	Strigil with 4 or 5 combs, each comb with more than 25 teeth (Fig. 3A, D)	*** Cenocorixa bui ***
–	Strigil with 4 or 5 combs, basal 2 combs with less than 5 teeth, apical combs with more than 25 teeth (Fig. 3B, E)	*** Cenocorixa crestiforma ***

#### Cenocorixa yuanjiangensis sp. nov.

Taxon classificationAnimaliaHemipteraCorixidae

E7333BC5-94A9-506B-B2AA-A9BA78157417

http://zoobank.org/88B196AD-A8AA-4000-AE62-9F5B58BC8345

Figures 1 , 2

##### Type material.

***Holotype*** ♂, **China**: Yuanjiang Wangxiangtai Nature Reserve [元江望乡台保护区], 23.38°N, 101.92°E, Yunnan Province, alt. 2020 m, 19.VII.2006, Ming LI leg. ***Paratypes***: same date as holotype, 3♂ 9♀.

**Figure 1.**
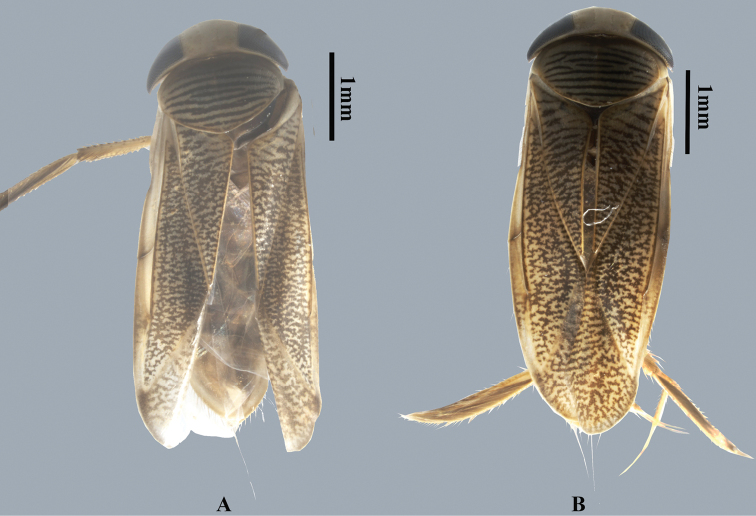
Dorsal habitus of *Cenocorixayuanjiangensis* sp. nov. **A** male **B** female.

##### Diagnosis.

**Figure 2.**
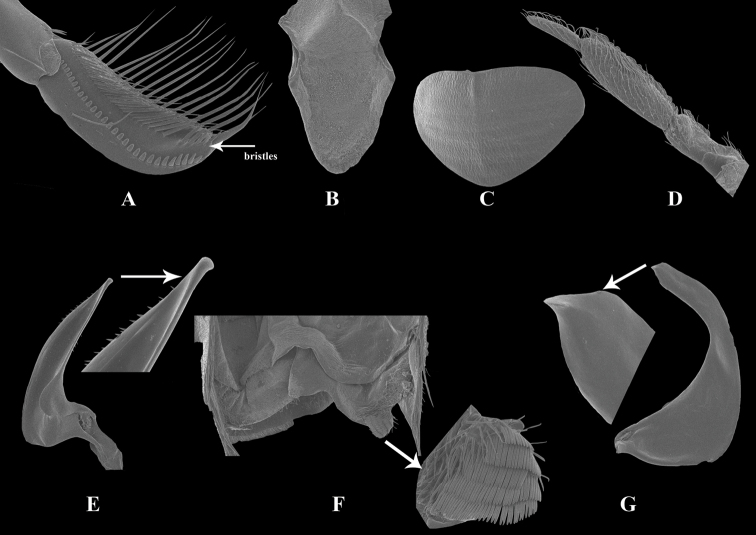
Male of *Cenocorixayuanjiangensis* sp. nov. **A** tibia **B** metaxyphus **C** pronotal carina **D** antenna **E** left paramere **F** seventh abdominal segment (strigil) **G** right paramere.

**Figure 3.**
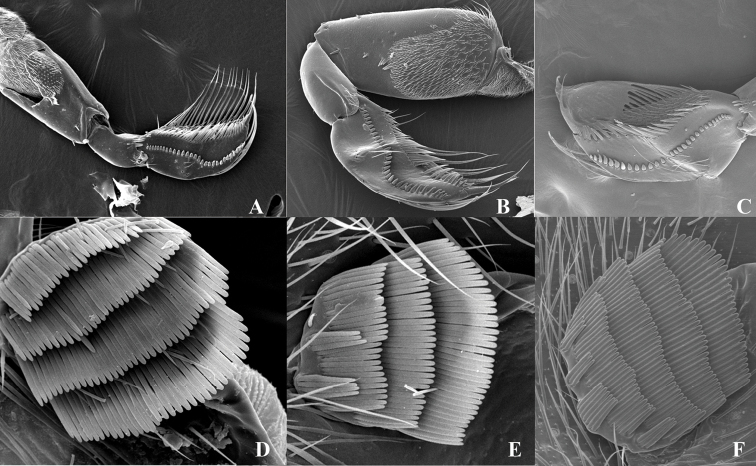
Male foreleg and strigil (Ren and Zhu 2010). **A–D***Cenocorixabui***B–E***Cenocorixacrestiforma***C–F***Cenocorixamontana*.

The new species is morphologically similar to *C.crestiforma* Ren & Zhu, 2010, from which it can be separated by the outline of the male pala (Figs 2A, 3B), the distinct pronotal carina on anterior part of pronotum, the feature of strigil (Figs 2F, 3E), and the right paramere (Fig. 2E, G). The *Cenocorixa* of China are somewhat smaller than species from the Nearctic, varying from 5.10 to 5.80 mm in length. The Nearctic *Cenocorixa* are distinguished by the presence of the abdominal strigil and by the narrower, more reticulate dark bands of the hemelytra, which tend to form longitudinal stripes (Lauck 1979).

##### Description.

Measurements: Male: Length of body 5.35 mm, width of body 1.72 mm; length of head 0.4 mm, width of head 1.72 mm, width of an eye 0.8 mm, interocular space 0.61 mm; length of pronotum 0.88 mm, width of pronotum 1.53 mm; length of pala 0.50 mm, width of median 0.17 mm; length of front tibia 0.33 mm, length of front femur 0.61 mm; length of segments of mesoleg: femur: tibia: tarsus: claw = 1.88: 0.91: 0.63: 0.80. length of forewing 4.50 mm, length of claval suture 2.11 mm, length of pruinose area of claval suture 0.82 mm; length of prenodal pruinose area of embolium 1.81 mm, length of postnodal pruinose area of embolium 1.02 mm. female: length of body 5.37 mm and width 2.02 mm; length of head 0.33 mm, width of head 1.88 mm, width of an eye 0.82 mm, interocular space 0.68mm; length of pronotum 0.70 mm, width of pronotum 1.58 mm; length of pala 0.59 mm, width of median 0.17 mm; length of front tibia 0.32 mm, length of front femur 0.62 mm; length of segments of mesoleg: femur: tibia: tarsus: claw = 1.81: 0.90: 0.62: 0.79; length of forewing 4.6 mm, length of claval suture 2.12 mm, length of pruinose area of claval suture 1.03 mm.

Moderately large and hairy, varying from 5.10 to 5.37 mm in length. Pattern on hemelytra consisting of numerous small, irregular, broken and anastomosing figures. Postocular space narrow; face depressed in male (Fig. 1A, B); median pronotal carina evident only on anterior third (Fig. 2C). Male pala broad, with longitudinal ridge on outside, at least basally; abdominal asymmetry dextral; strigil moderately large (Fig. 2F).

##### Color.

Body brown, with yellowish patterns (Fig. 1); transverse bands on pronotum yellow; corium and membrane separated by a pale line; Basal 3 segments brown except lateral and posterior margin in male whereas abdomen ventral yellowish in female.

Vertex of head rounded and broad in both sexes; 7–9 bands on pronotum, sometimes discontinuous, equal or slightly wider than dark interspaces; lateral angle of pronotum blunt; lateral lobes of prothorax narrow, sides almost parallel, apex rounded; pala of male broad, with inconspicuous ridge across face, bearing a curving peg row consisting of 28–29 pegs, distal 5–7 pegs longer and spiculate (Fig. 2A); upper palmar bristles separated into two parts, 5 anterior and 26 posterior (Fig. 2A); metaxyphus triangular, base narrower than long; hemelytron with vermiculate patterns but irregular at apex of clavus (Fig. 2B); antennae with four segments, segment I–II thick and short, segment III thick and longest, segment IV slender and half the length of segment III (Fig. 2D); strigil quadrate consisting of 5 combs (Fig. 2F); median setiferous lobe of 7^th^ abdominal tergite narrow and curved, apex blunt(Fig. 2F); right paramere strongly curved at apical 1/3, with a small pointed lobe at tip (Fig. 2G); left paramere of male as shown in Figure 2E.

##### Distribution.

**Figure 4.**
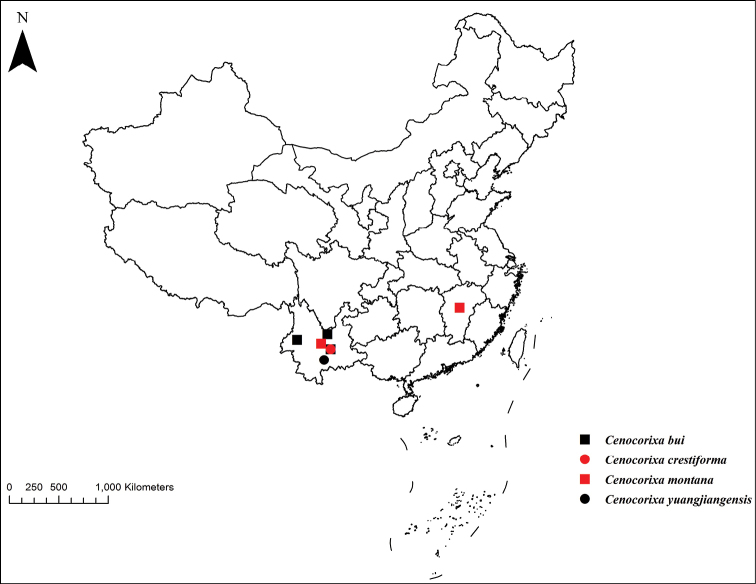
Distribution of *Cenocorixa* within China.

Currently known only from Yunnan Province, China (Fig. 4).

##### Etymology.

Referring to Yuanjiang County in which the type locality situated.

## Supplementary Material

XML Treatment for Cenocorixa yuanjiangensis
